# Polycyclic Guanidine Alkaloids from Poecilosclerida Marine Sponges

**DOI:** 10.3390/md14040077

**Published:** 2016-04-09

**Authors:** Estelle Sfecci, Thierry Lacour, Philippe Amade, Mohamed Mehiri

**Affiliations:** 1 Nice Institute of Chemistry, Marine Natural Product Team, University Nice Sophia Antipolis, Parc Valrose, 28 avenue de Valrose, 06108 Nice Cedex 02, France; sfecci@unice.fr (E.S.); amade@unice.fr (P.A.); 2 Biopreserv, 4 traverse Dupont, 06130 Grasse, France; tlacour@biopreserv.fr

**Keywords:** marine sponges, Poecilosclerida, alkaloids, crambescidins, batzelladines, analytical tools, bioactivity

## Abstract

Sessile marine sponges provide an abundance of unique and diversified scaffolds. In particular, marine guanidine alkaloids display a very wide range of biological applications. A large number of cyclic guanidine alkaloids, including crambines, crambescins, crambescidins, batzelladines or netamins have been isolated from Poecilosclerida marine sponges. In this review, we will explore the chemodiversity of tri- and pentacyclic guanidine alkaloids. NMR and MS data tools will also be provided, and an overview of the wide range of bioactivities of crambescidins and batzelladines derivatives will be given.

## 1. Introduction

The most primitive benthic marine organisms are also among the most chemodiversified producers of secondary metabolites (SM). These compounds are used for predation and competition for space, as well as for communication, and protection against potential surrounding aggressors. Due to the high water dilution, the produced metabolites exhibit several potent biological activities [1]. Several reviews dealing with bioactive marine natural products, in particular, alkaloids have been published in the last few decades [2,3,4,5,6,7,8,9,10,11,12,13,14,15,16,17,18,19]. Of the many different marine species that have been researched, sponges are likely to be the most studied sources of marine natural products due to the large amount of structurally diverse SM scaffolds they produce. Even so, the last few years have seen a slight decline in the number of newly described metabolites [17,18].

In this review, we focused on the chemodiversity and biological activities of batzelladine- and crambescidin-like guanidine alkaloids isolated from Poecilosclerida marine sponges. These polycyclic guanidine alkaloids are extremely versatile SM [20]. Since their original isolation in 1989, several revisions have been made and many aspects remain to be studied. However, what is clear is that they seem to be specific to Poecilosclerida marine sponges. Over 53 derivatives have been isolated and their original structures, based on chemical degradation studies and extensive NMR and MS studies, have been revised since the development of their total synthesis. The structural and stereochemical complexity of this class of natural products have inspired the development of a number of new synthetic methodologies [21,22,23,24,25,26,27,28,29,30,31,32,33,34], which in turn has led to several total syntheses [31,33,35,36,37,38,39,40,41,42,43,44,45,46,47,48,49,50] beyond the scope of this review. Finally, these unique and fascinating structures are coupled with a wide range of biological activities due to the typical shape of their tricyclic skeleton. However, little is known about their exact mechanism of action.

## 2. Structural Diversity

From 1989 to 2015, 53 TGA (triazaacenaphthylene guanidine alkaloids) have been isolated. Natural TGA reported to date are listed in Table 1 and Table 2. 

Ptilomycalin A (**1**), isolated from the sponges *Ptilocaulis spiculifer* and *Hemimycale* sp. [51], is the parent member of a group of related metabolites. This includes crambescidins [52,53,54,55] and their derivatives (isocrambescidine (**6**) [52,56], crambidine (**7**) [52], neofolitispates (**8**–**10**) [57], crambescidin acid (**14**) [58], crambescidic acid (**15**) [59], ptilomycalin D (**17**) [60], monanchocidins (**18**–**22**) [61,62], and monanchomycalins (**23**–**25**) [63,64]), which are found in several sponges (*Crambe crambe*, *Neofolitispa dianchora, Monanchora arbuscula*, *Monanchora ungiculata,* *Monanchora dianchora*, and *Monanchora pulchra*), as well as, for a subset, in the New Caledonian starfishes *Fromia monilis* and *Celerina heffernani* (Table 1) [65]. Additional guanidine derivatives have been isolated, such as the batzelladines A–E (**26**–**30**) from the Bahamanian sponge *Batzella* sp. (which has since been revised to *Crambe crambe*) [66], batzelladines F–I (**31**–**34**) from the Jamaican sponge *Batzella* sp. [67], batzelladine J (**51**) from the Caribbean sponge *Monanchora unguifera* [59], and their derivatives (clathriadic acid (**41**) [68], merobatzelladines (**42**,**43**) [69], and batzellamide A (**50**) [70]) from *Monanchora unguifera*, *Clathria calla*, and *Monanchora arbuscula* (Table 2). 

**Figure 1:**
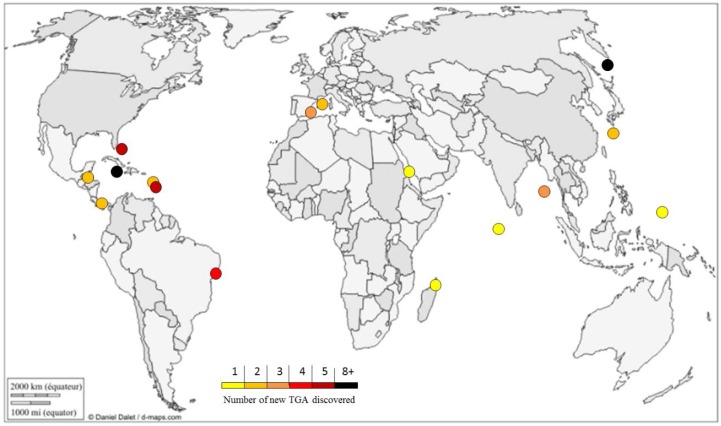
Geographical repartition of new TGA (triazaacenaphthylene guanidine alkaloids) discovered from 1989 to 2015. Graphic ^©^ d-maps.com [72]. Used with permission.

TGA are only found in Poecilosclerida marine sponges. To date, they have been only isolated from the Crambidae *(Crambe*, *Monanchora*), Mycalidae *(Arenochalina*, *Mycale*), Phoriospongidae *(Batzella*, *Hemimycale*), and Microcionidae (C*lathria*) families [71]. Sponges that produce TGA can be found in warm waters, without distinction between oceans and seas. A large majority were sampled in the Caribbean Sea (Figure 1).

**Figure 2:**
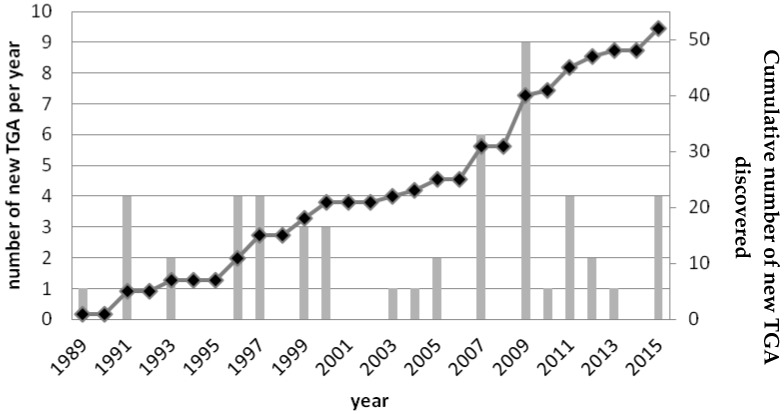
Number of new TGA discovered since 1989. The number of TGA discovered per year (grey bars) and the cumulative data representing the total number of TGA discovered (black curve) are presented from 1989 to 2015.

Since 1989, the number of new TGA discovered each year is considered as almost constant, although it should be noted that in some years no new TGA were described (Figure 2).

### 2.1. TGA Structures

Each TGA is structurally closely related to the others. Crambescidins-like GA differ from one another, by the C-8 spiro ring, the presence or absence of a hydroxyl group at C-13 or the nature of the side chain terminus at C-14. 

**Figure 3:**
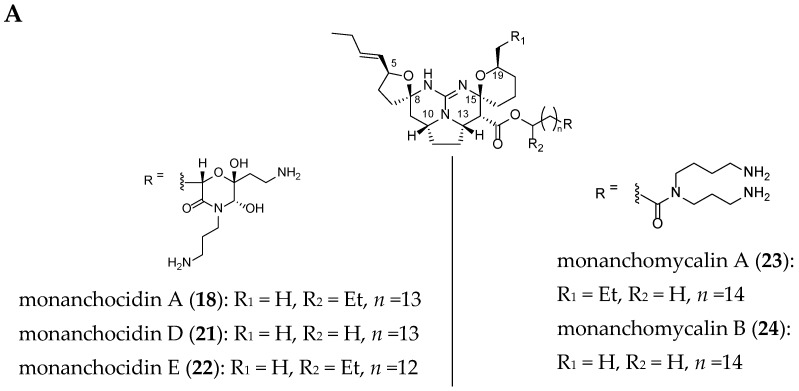

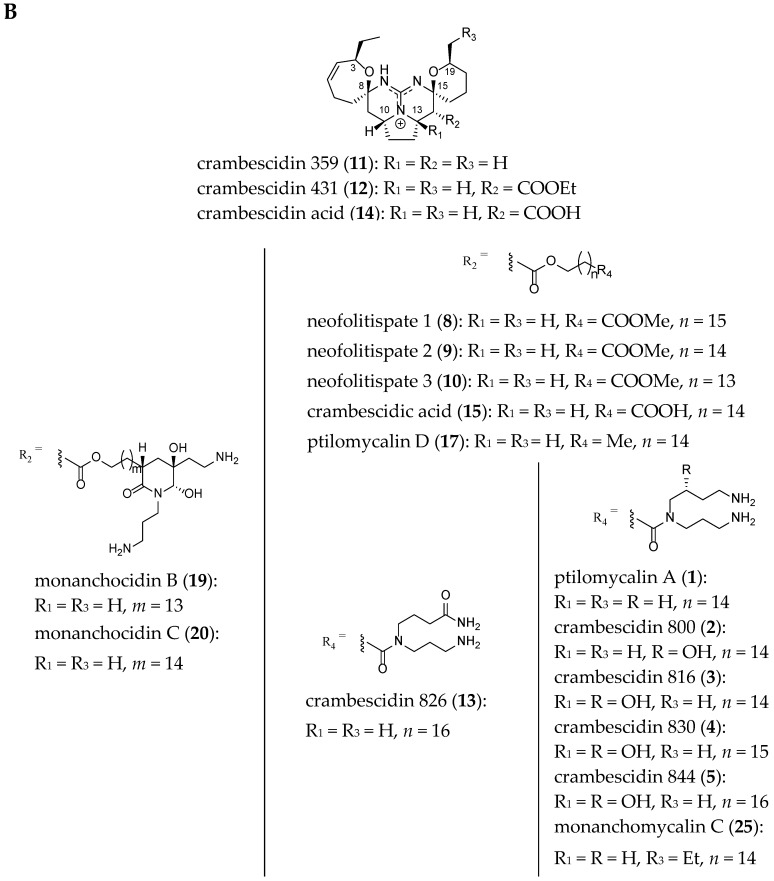
Related (**A**) C-8 cycloheptanic spiro ring and (**B**) C-8 cyclopentanic spiro ring crambescidin-like GA.

The structures of 22 related crambescidin-like GA are summarized in Figure 3.

**Figure 4:**
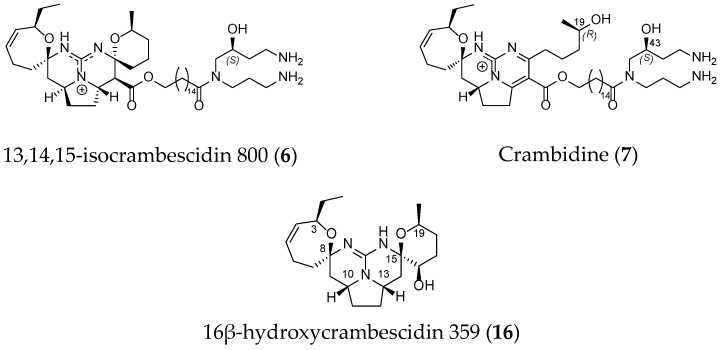
Non-related crambescidin-like GA.

Other derivatives such as 13,14,15-isocrambescidin 800 (**6**), crambidin (**7**) or 16β-hydroxycrambescidin 359 (**16**) have also been isolated (Figure 4).

**Figure 5:**
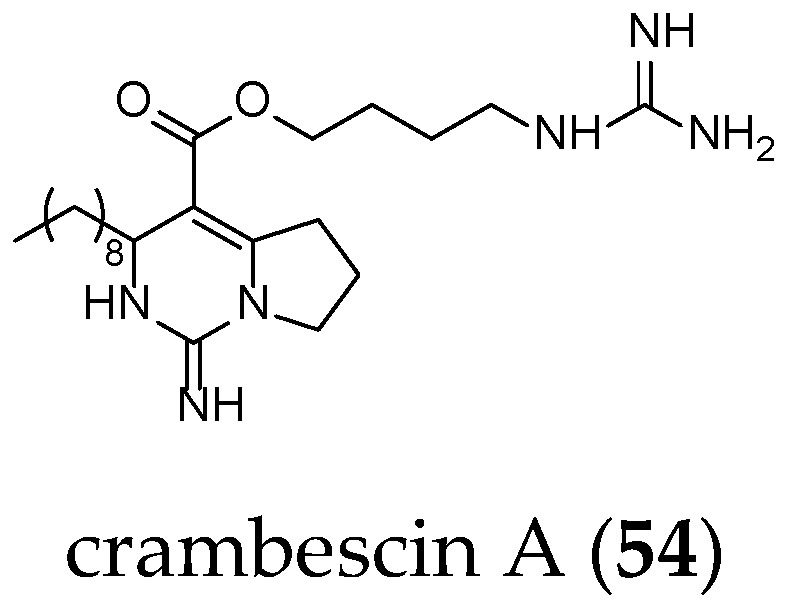
Crambescin A (**54**).

Compared to crambescidin-like GA, batzelladines-like GA present more structural variations. Structurally, all of the batzelladines share a decahydro- or octahydro-5,6,8b-triazaacenaphthalene core with different degrees of oxidation. Batzelladines A–I (**26**–**34**) all possess at least one tricyclic guanidine core that contains either a *syn* or *anti* stereo relationship of the angular hydrogens that flank the pyrrolidine nitrogen [76]. To this, tricyclic guanidine core are connected, through an ester linkage, and additional guanidine fragments of varying complexity. Batzelladines F (**31**), G (**32**), and L (**38**) all possess an additional tricyclic hydroxy-guanidine fragment, while the simplest members of the family, batzelladines C (**28**), D (**29**), and E (**30**), possess a common 4-guanidino-butyl unit. The more complex batzelladines, A (**26**) and B (**27**), are attached to an analogue of crambescin A (**54**) (Figure 5). Batzelladines F (**31**) and J (**36**) are composed of two 5,6,6a-triaza-acenaphthalene cores linked via an aliphatic chain.

The structures of several members of the batzelladines have been revised since their original isolation. The originally proposed structures were based on chemical degradation studies, NMR spectroscopy analysis (1D and 2D), and comparison of the data to previously reported polycyclic guanidine such as ptilomycalin A (**1**). Since the original isolation work, the structures of batzelladines A (**26**), D (**29**), E (**30**), and F (**31**) have all been revised to their current structures after their partial or total synthesis [27,28,29,30,35,39]. As a consequence of these reassignments, the relative stereochemistry of batzelladines G (**32**), H (**33**), and I (**34**) has been reexamined [30].

**Figure 6:**
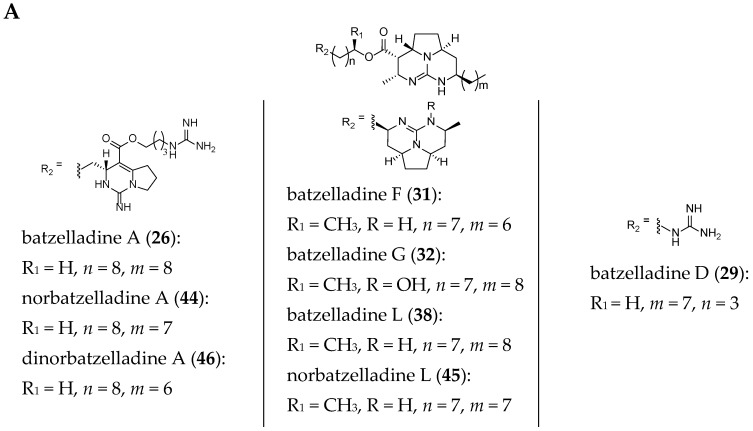

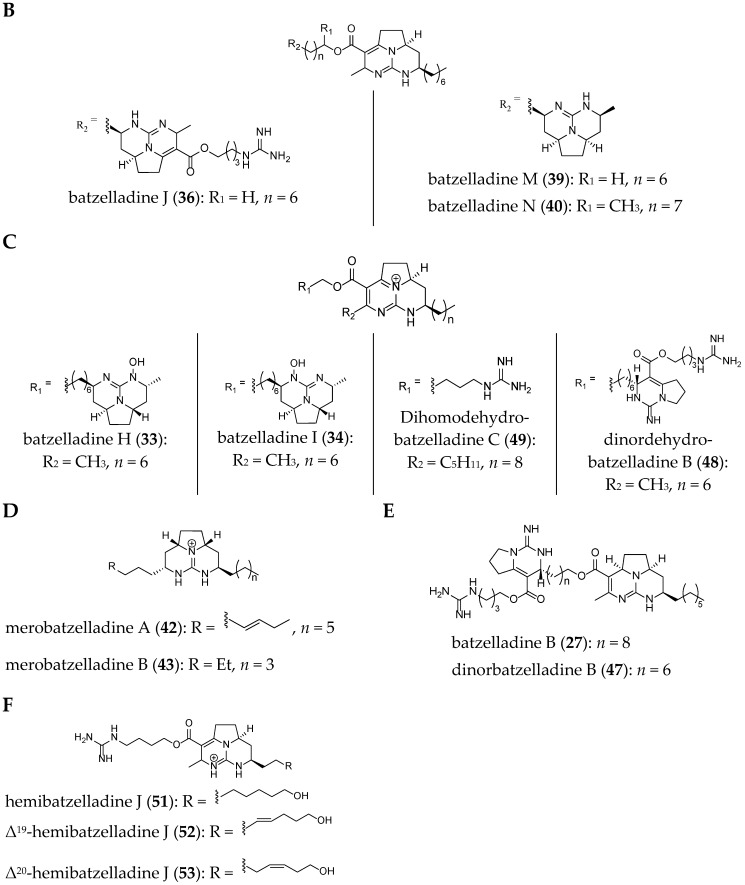
Related batzelladine-like GA with different right-handed tricycles.

The current structures of 22 related batzelladine-like GA are summarized in Figure 6.

**Figure 7:**
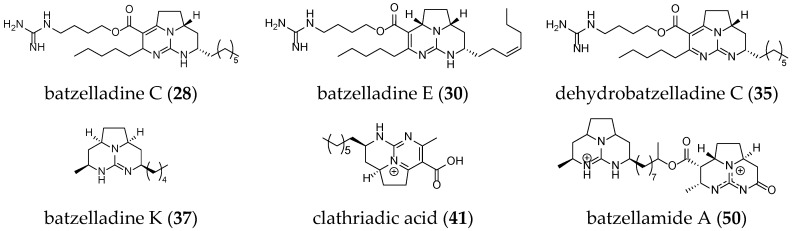
Unrelated batzelladine-like GA.

Other batzelladine-like GA have been isolated such as batzelladine C (**28**), K (**37**), and E (**30**); dehydrobatzelladine C (**35**); clathriadic acid (**41**); and batzellamide A (**50**) (Figure 7).

In summary, the structurally unique tricyclic guanidinium ring system (hydro-5,6,6atriazaacenapthalene) that defines this class of natural products can be found in over 53 different alkaloids. While each of these natural products share this common structural motif, the substituents around the tricyclic core of these molecules leads to a significant structural diversity, which relate to a wide-range of biological properties. For instance, a large number of these molecules including batzelladine F (**31**) and ptilomycalin A (**1**) feature esters that tether the tricyclic core to a diverse array of different functional groups, including other tricyclic guanidine subunits. The other area of structural diversity within this family of alkaloids is both the C1 and the C8 alkyl chains, which vary in terms of length, units of unsaturation, and oxidation. Remote oxidation of the alkyl branches is characteristic of the crambescidin alkaloids, including ptilomycalin A (**1**) and crambescidin 359 (**11**), which feature two spirocyclic hemiaminals as well as the tricyclic guanidine framework. In addition to structural diversity, batzelladines-like GA and crambescidins-like GA alkaloids feature different stereochemical configurations of the tricyclic core. Both the *trans*- and *cis*-configurations of the pyrrolidine subunit have been reported in this class of natural products. Batzelladine F (**31**) highlights this stereochemical diversity as it contains two distinct tricyclic guanidine subunits, with each featuring one of the pyrrolidine configurations. The stereochemical diversity is generally limited to the configuration of the pyrrolidine unit, as all of the alkaloids in this class feature a *trans* relationship between the C4 (and/or C6) proton and the C1 (and/or C8) alkyl chain, with the exception of merobatzelladines A (**42**) and B (**43**). Both natural products feature a *cis* relationship between the C6 proton and the C8 alkyl chain. 

### 2.2. TGA Classification

**Figure 8:**
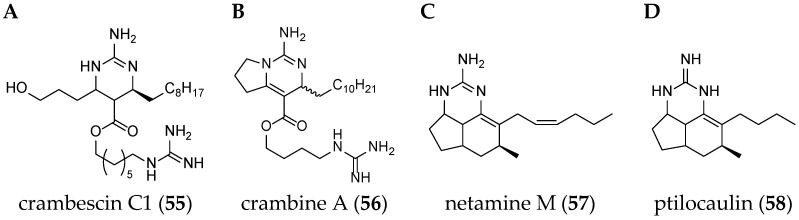
(**A**) Crambescin C1 (**55**); (**B**) crambine A (**56**); (**C**) netamine M (**57**); and (**D**) ptilocaulin (**58**).

Different guanidine alkaloids classifications can be made, and, as such, Santos *et al.* (2015) have described four GA chemotypes [70]. The first class is constituted of a monocyclic pyrimidinamine skeleton, for example, crambescin C1 (**55**) or the bicyclic cyclopentapyrimidinamine skeleton, such as crambine A (**56**) (Figure 8). The second one is a tricyclic triazaacenaphthylene skeleton, which only contains one guanidine moiety like crambescidins, and the third one possess the same skeleton as for class 2, but contains, at least, one more guanidine moiety, as can be seen in the case of most batzelladines. The fourth one is a tricyclic cyclopentaquinazolinamine skeleton like netamine M (**57**) or ptilocaulin (**58**).

Another classification can be made with the same first, second and fourth classes, and a modified third class describing pentacyclic triazaacenaphthylene skeletons like crambescidins (**1**–**25**) (Table 3). 

Without taking into account any genus revision made, some observations may be outlined. According to the data reported, *Monanchora* is the most studied genus (Table 1 and Table 2). Some species seem to produce both the crambescidins and batzelladines classes as is illustrated with *Monanchora unguifera*, which produces 16β-hydroxycrambescidin (**16**), crambescidic acid (**15**) and batzelladines J–L (**36**–**38**) (Table 1 and Table 2). Other genera seem to produce only one TGA family. For example, to date, *Batzella* sp. was only shown to produce batzelladines A–I (**26**–**34**).

Interestingly, a chemotaxonomic study suggested that the biogenetically related guanidine alkaloids isolated from *Crambe crambe*, *Monanchora arbuscula*, *Ptilocaulis spiculifer*, and *Hemimycale* sp., should eventually be united in a single genus, preferentially *Crambe* [53].

### 2.3. Analytical Tools for TGA Structural Analysis

TGA structural determinations have been accomplished based on extensive NMR (1D and 2D) and MS analyses, and chemical degradation. 

The following section is a guideline for the structure determination of crambescidin- or batzelladine-like GA derivatives by their characteristic fragments via the use of NMR and MS analyses.

#### 2.3.1. NMR Spectroscopy

**Figure 9:**
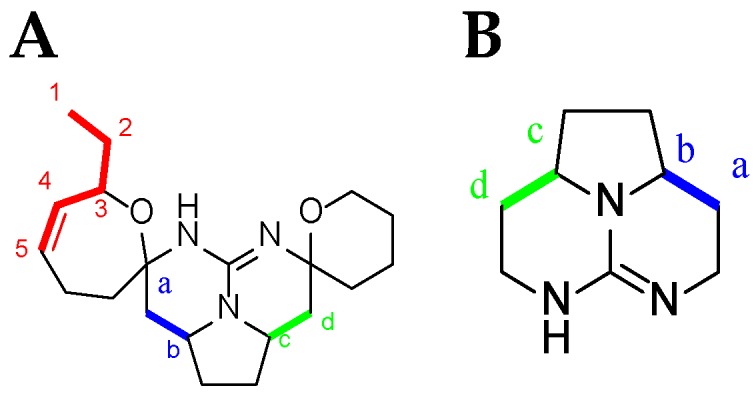
Current numbering scheme for: (**A**) C-8 cycloheptanic spiro ring crambescidin-like GA; and (**B**) batzelladine-like GA.

TGA NMR spectra have a high content of information. Due to the relative signal richness, the ^1^H–^1^H COSY experiment is appropriate in helping to find whether one or another TGA class is present by analyzing the NMR spectra between 0.8 and 5 ppm. Nonetheless, several characteristic signals from different atoms listed below (Figure 9) should be found in the ^1^H NMR spectra (Table 4).

##### Crambescidin Case: Ptilomycalin A (**1**)

As an example, we choose ptilomycalin A (**1**), which is part of the pentacyclic TGA class.

**Figure 10:**
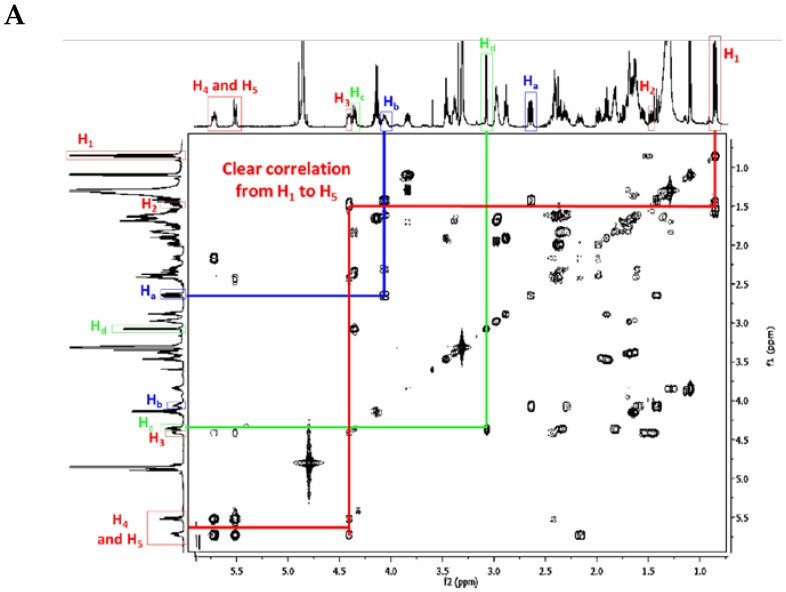

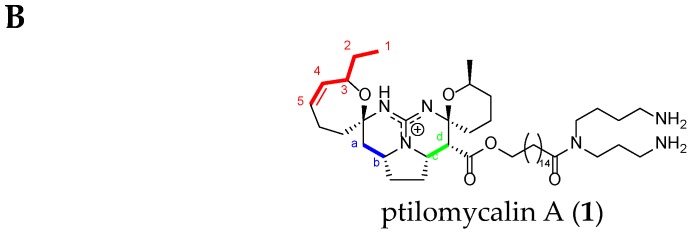
(**A**) Ptilomycalin A (**1**) ^1^H-^1^H COSY NMR spectrum in CD_3_OD (personal data); and (**B**) labeled ptilomycalin A (**1**).

First of all, the H_1_ signal (triplet) and the H_4_ and H_5_ signals of the double bond are very characteristic. There are also clear correlations from H_1_ to H_5_, and finally, the NMR chemical shifts of H_a_, H_b,_ H_c,_ and H_d_ are also very characteristic within the crambescidin family (Figure 10) (Table 4).

##### Batzelladine Case: Batzelladine F (**31**)

In general, all the NMR signals reported in Table 4 are more shielded in batzelladines than in crambescidins. Moreover, the NMR spectra are more complex and are often different since the guanidinium core can be once or more dehydrogenated.

Within the tricyclic TGA class 2, we used the example of batzelladine F (**31**).

**Figure 11:**
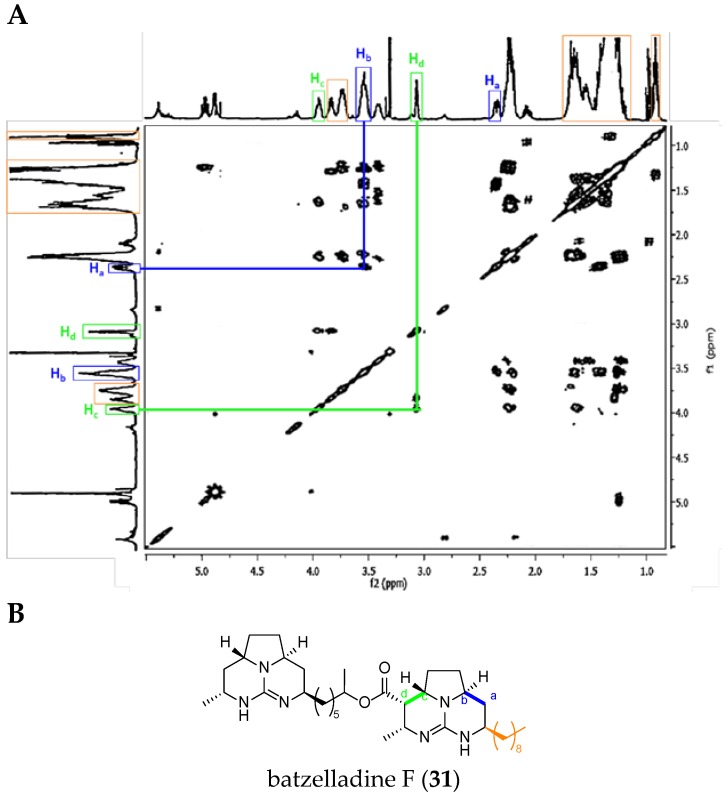
(**A**) Batzelladine F (**31**) ^1^H-^1^H COSY NMR spectrum in CD_3_OD (adapted from Patil *et al.* [67]); and (**B**) labeled batzelladine F (**31**).

The major difference between batzelladine F and ptilomycalin A spectra is the absence of the signals for the protons H_4_ and H_5_, and the corresponding correlation. On the other hand, two correlations are very characteristic within batzelladines: H_a_ with H_b_, and H_c_ with H_d_ (Figure 11).

#### 2.3.2. Mass Spectrometry

Several TGA were detected by positive electrospray mass spectrometry ionization studies. Usually, authors notify the TGA quasi-molecular ion [M + H]^+^ with the exception of batzelladines M (**39**) and N (**40**), which were detected through their dicharged ion [M + 2H]^2+^ [73]. On the other hand, both quasi-molecular and discharged ions were observed for batzelladine L (**38**) and monanchomycalin C (**25**). Mass spectrometry data for TGA are summarized in Table 5. 

Tandem mass spectrometry (MS²) experiments were also performed to confirm a hypothesis or provide additional information concerning the TGA side chains, as reported in Table 6.

## 3. Biological Activities

TGA exhibited a wide range of biological activities with mainly antiviral, antimicrobial (including antifungal, antibacterial, anti-yeast, and antiparasitic), and antitumor properties.

### 3.1. Antiviral Activities

Several TGA derivatives were evaluated for their antiviral activities against different viruses such as Human immunodeficiency virus (HIV-1), *Herpes simplex* virus (HSV-1), and Human hepatitis B virus (HBV). The reported data for 17 TGA are summarized in Table 7. 

In general, crambescidin-like GA seem to be more efficient against HIV compared to batzelladine-like GA (half maximal effective concentration (EC_50_) around 1 mM [66,73]) with EC_50_ activities below 0.05 µM [51,73]. The stereochemistry of the molecule has a great influence on the antiviral activity. For example, 13,14,15-isocrambescidin (**6**) is not active against HSV-1 compared to crambescidins [56]. Ptilomycalin A (**1**) shows very potent anti-HIV-1 and anti-HSV-1 activities at a concentration of 0.011 and 0.25 µM, respectively [51], which makes it the best antiviral candidate. 

Surprisingly, neofolitispates (**8**–**10**) are the only crambescidin-like GA derivatives that have been tested on HBV and exhibited an anti-HBV activity [57]. Unfortunately, few details have been reported.

On the other hand, batzelladines A–E (**26**–**30**) were shown to block the interaction between the surface of the HIV envelope glycoprotein gp120 and the extracellular domains of human CD4 receptor protein [77]. This binding is vital to the replication of the virus as it controls its entry into the human cells, since without access to the biochemical environment within the cell the virus is unable to replicate. As a consequence, batzelladines have a therapeutic interest in the treatment of HIV [76].

To date, batzelladine-like GA have not been tested against HSV or HBV viruses. 

### 3.2. Antimicrobial Activities

TGA derivatives were tested against several bacteria (*Mycobacterium tuberculosis*, *Staphylococcus aureus*, *Pseudomonas aeruginosa*, and *Mycobacterium intracellulare*), yeast (*Candida albicans*), fungi (*Cryptococcus neoformans* and *Aspergillus fumigatus*), and parasites (*Plasmodium falciparum*, *Tripanosoma cruzi*, *Leishmania infatum*, and *Trypanosoma brucei brucei*). Table 8 reports all the antimicrobial activity results for 19 TGA tested. 

Crambescidin 800 (**2**) and ptilomycalin A (**1**) exhibited potent activity against most bacteria, yeast, fungi, and parasites [68,73]. Curiously, they are poorly active on *Mycobacterium intracellulare* and are considered inactive on *Mycobacterium tuberculosis* (*M. tuberculosis*) [73]. Furthermore, 16β-hydroxycrambescidin (**16**), the only crambescidin-like GA bearing a hydroxyl group on its pentacycle, was not active against all the strains tested [73].

In some cases, Batzelladine-like GA present similar activities compared to crambescidin-like GA. Batzelladines C (**28**) and L (**38**) are often the more potent molecules [73]. Nevertheless, batzelladine M (**39**) is the less active TGA class 2 and surprisingly, the same authors showed batzelladine N (**40**) to be nine times more efficient compared to batzelladine M (**39**) against *M. tuberculosis* (minimum inhibitory concentration (MIC) 3.18 and 28.5 µg·mL^−1^, respectively), despite their chemical structures being closely related [73].

### 3.3. Antitumoral Activities

Over 20 TGA were tested for their cytotoxicity against several cancer cell lines such as prostate, ovary, breast, melanoma leukemia, pancreas, colon, and cervix (Table 9).

Ptilomycalin A (**1**) is once again the most active TGA tested as its half maximal growth inhibition values (GI_50_) are always below 0.1 µg/mL on all the cell lines tested [73]. Crambescidin 800 (**2**) shows similar activities [73]. Compared to TGA class 3, TGA class 2 is less active, although batzelladine C (**28**) and L (**38**) and dehydrobatzelladine C (**35**), in general, exhibited a GI_50_ below 1 µg/mL [73]. Finally, crambescidin 816 (**3**) was shown to inhibit cell migration by altering the cytoskeleton dynamics and induced cell death by apoptosis [75,78].

In summary, both TGA class 2 and 3 have been tested against viruses, microbes, and several cancer cell lines. The wide-ranging biological activity of this class of natural products can be in part attributed to the cationic nature of the guanidinium functional group that can participate in a large number of non-covalent molecular interactions [77]. In general, TGA from class 3 exhibited better activities compared to TGA from class 2 [73]. Within TGA class 3, ptilomycalin A (**1**) and crambescidin 800 (**2**) showed similar activities in all assays, suggesting that the hydroxyl group of the right-handed portion in crambescidin 800 (**2**) did not affect the bioactivity. Nevertheless, 16β-hydroxycrambescidin 359 (**16**) did not show any significant antimicrobial activity, suggesting that the hydroxyl group, located on the pentacycle at C-16, diminished the activity [73]. Further studies are needed to confirm this hypothesis. This could be assessed by evaluating the inhibitory activities of crambescidins 816 (**3**), 830 (**4**) and 844 (**5**), as they carry the same hydroxyl moiety as crambescidin 800 (**2**) and another hydroxyl located in the pentacycle at C-13. In parallel, several batzelladines have been reported to disrupt protein–protein interactions. Elucidation of the mechanism by which protein–protein interactions are modulated by these molecules is of great interest, since protein–protein associations are important in all the aspects of cell biochemistry. Moreover, small molecules that influence protein–protein association would be new biological tools and potential therapeutic agents. In particular, batzelladines or their derivatives may prove to be applicable for AIDS treatment. Batzelladines A–E (**26**–**30**) block interaction between the surface of the HIV envelope glycoprotein gp120 and the extracellular domains of human CD4 receptor protein [66]. A subset of batzelladines exhibited also potential immunosuppressive activity as they induce dissociation of the complex between the protein kinase p56^lck^ and CD4 [67]. Synthetic derivatives of batzelladines were reported to disrupt Nef–p53, Nef–actin, and Nef–p56^lck^ interactions [77]. Bewley *et al.* tested a series of 28 synthetic batzelladine-like GA analogues on HIV-1 cell fusion assay, to find structure-activity relationships [76]. According to this study, the greater the rigidity of the molecule, the less biologically active it is. Moreover, they have shown that the most active compounds tested were compounds which contain two tricyclic guanidine moieties connected by an alkyl ester linkage including eight heavy atoms, such as batzelladines F (**31**) and G (**32**). Batzelladines biological properties could be dramatically affected by the ester side chains. For example, batzelladine A (**26**) inhibits the binding of HIV glycoprotein gp120 to CD4 receptors, whereas batzelladine D (**29**) has no known biological activity [77].

Finally, several derivatives that do not feature an ester side chain may still exhibit interesting biological properties such as the antibacterial and antimalarial activities of merobatzelladines A (42) and B (43) [69].

## 4. Conclusions

Several batzelladine- and crambescidin-like guanidine alkaloids have been isolated from Poecilosclerida marine sponges. Their biosynthesis, or the biosynthesis of their precursors, may involve symbiotic microorganisms [79]. This class of natural products is structurally unique as all the derivatives are constituted by a tricyclic guanidinium ring system, to which are appended different substituents. This significant structural diversity has led to wide-ranging biological properties with mainly antiviral, antimicrobial (including antifungal, antibacterial, anti-yeast, and antiparasitic), and antitumor activities. In addition to the biomimetic strategy, a number of groups completed the total synthesis of several of these alkaloids by developing new synthetic methodologies. Although there are many efficient and stereoselective synthetic routes towards this class of natural products, there is not yet one method that would allow entry into all of the cores of this family. Nonetheless, a number of these syntheses have enabled the establishment of their relative and absolute stereochemical configurations. In conclusion, several of these alkaloids and their synthetic analogs were prepared on a large enough scale to allow further biological testing, including few structure-activity relationship studies. Ongoing studies may provide us with further clues regarding this class of Marine Natural Products.

## Figures and Tables

**Table 1:** Reviewed crambescidin-like GA (guanidine alkaloids) from 1989 to 2015.

Metabolite	Species	Sampling Site	Discovery Year	Guanidine Moiety	Biological Activity	Synthesis Described	Ref.
ptilomycalin A (**1**)	*Hemimycale* sp. *Ptilocaulis spiculifer*	Red sea	1989	1	Av, Am, At	Yes	[42,51,73]
crambescidin 800 (**2**)	*Crambe crambe*	Palma de Mallorca, Mediterranean sea	1991	1	Av, Am, At	Yes	[54,55,56,73,74]
crambescidin 816 (**3**)	*Crambe crambe*	Palma de Mallorca, Mediterranean sea	1991	1	Av, At, Ca^2+^ antagonist	No	[52,55,56,75]
crambescidin 830 (**4**)	*Crambe crambe*	Palma de Mallorca, Mediterranean sea	1991	1	n.t.	No	[55]
crambescidin 844 (**5**)	*Crambe crambe*	Palma de Mallorca, Mediterranean sea	1991	1	Av	No	[55,56]
13,14,15-isocrambescidine 800 (**6**)	*Crambe crambe*	Banyuls, Mediterranean sea	1993	1	Not active	Yes	[36,42,56]
crambidine (**7**)	*Crambe crambe*	Banyuls, Mediterranean sea	1993	1	n.t.	Yes	[45,49,52]
neofolitispate 1 (**8**)	*Neofolitispa dianchora*	Andaman Islands, Indian Ocean	1999	1	Av	No	[57]
neofolitispate 2 (**9**)	*Neofolitispa dianchora*	Andaman Islands, Indian Ocean	1999	1	Av	Yes	[57]
neofolitispate 3 (**10**)	*Neofolitispa dianchora*	Andaman Islands, Indian Ocean	1999	1	Av	No	[57]
crambescidin 359 (**11**)	*Monanchora ungiculata*	Belize, North Atlantic Ocean	2000	1	n.t.	Yes	[33,41,48,53]
crambescidin 431 (**12**)	*Monanchora ungiculata*	Belize, North Atlantic Ocean	2000	1	n.t.	No	[53]
crambescidin 826 (**13**)	*Monanchora* sp.	Palau, Pacific Ocean	2003	1	Av	No	[54]
crambescidin acid (**14**)	*Monanchora ungiculata*	Maldive Islands, Indian Ocean	2004	1	At	No	[58]
crambescidic acid (**15**)	*Monanchora unguifera Monanchora dianchora*	Panama, Caribbean side, Atlantic Ocean	2005	1	n.t.	No	[59]
16β-hydroxycrambescidin 359 (**16**)	*Monanchora unguifera*	Jamaica, North Atlantic Ocean	2007	1	Am	No	[73]
ptilomycalin D (**17**)	*Monanchora dianchora*	Madagascar, Indian Ocean	2007	1	n.t.	No	[60]
monanchocidin A (**18**)	*Monanchora pulchra*	Urup Island, North Pacific Ocean	2010	1	At	No	[61]
monanchocidin B (**19**)	*Monanchora pulchra*	Urup Island, North Pacific Ocean	2011	1	At	No	[62]
monanchocidin C (**20**)	*Monanchora pulchra*	Urup Island, North Pacific Ocean	2011	1	At	No	[62]
monanchocidin D (**21**)	*Monanchora pulchra*	Urup Island, North Pacific Ocean	2011	1	At	No	[62]
monanchocidin E (**22**)	*Monanchora pulchra*	Urup Island, North Pacific Ocean	2011	1	At	No	[62]
monanchomycalin A (**23**)	*Monanchora pulchra*	Urup Island, North Pacific Ocean	2012	1	At	No	[63]
monanchomycalin B (**24**)	*Monanchora pulchra*	Urup Island, North Pacific Ocean	2012	1	n.t.	No	[63]
monanchomycalin C (**25**)	*Monanchora pulchra*	Urup Island, North Pacific Ocean	2013	1	n.t.	No	[64]

Am, antimicrobial activity; Av, antiviral activity; At, antitumoral activity; and n.t., not tested.

**Table 2:** Reviewed batzelladine-like GA from 1989 to 2015.

Metabolites	Species	Sampling Site	Discovery Year	Guanidine Moiety	Biological Activities	Synthesis Described	Ref.
batzelladine A (**26**)	*Batzella* sp.	Bahamas, North Atlantic Ocean	1996	3	Am, Av	Yes	[32,46,66]
batzelladine B (**27**)	*Batzella* sp.	Bahamas, North Atlantic Ocean	1996	3	Av	No	[66]
batzelladine C (**28**)	*Batzella* sp.	Bahamas, North Atlantic Ocean	1996	2	Av, Am, At	No	[66,73]
batzelladine D (**29**)	*Batzella* sp.	Bahamas, North Atlantic Ocean	1996	2	Av, Am	Yes	[37,40,46,47,66]
batzelladine E (**30**)	*Batzella* sp.	Bahamas, North Atlantic Ocean	1996	2	n.t.	Yes	[35,66]
batzelladine F (**31**)	*Batzella* sp.	Jamaica, North Atlantic Ocean	1997	2	Am	Yes	[30,39,42,67]
batzelladine G (**32**)	*Batzella* sp.	Jamaica, North Atlantic Ocean	1997	2	Av	No	[67]
batzelladine H (**33**)	*Batzella* sp.	Jamaica, North Atlantic Ocean	1997	2	Av	No	[67]
batzelladine I (**34**)	*Batzella* sp.	Jamaica, North Atlantic Ocean	1997	2	Av	No	[67]
dehydrobatzelladine C (**35**)	*Monanchora arbuscula*	Belize, North Atlantic Ocean	2000	2	Av, Am, At	Yes	[43,53,73]
batzelladine J (**36**)	*Monanchora unguifera*	Panama, North Atlantic Ocean	2005	3	n.t.	No	[59]
batzelladine K (**37**)	*Monanchora unguifera*	Jamaica, North Atlantic Ocean	2007	1	n.t.	No	[73]
batzelladine L (**38**)	*Monanchora unguifera*	Jamaica, North Atlantic Ocean	2007	2	Av, Am, At	No	[73]
batzelladine M (**39**)	*Monanchora unguifera*	Jamaica, North Atlantic Ocean	2007	2	Av, Am, At	No	[73]
batzelladine N (**40**)	*Monanchora unguifera*	Jamaica, North Atlantic Ocean	2007	2	Av, At	No	[73]
clathriadic acid (**41**)	*Clathria calla*	Martinique, North Atlantic Ocean	2009	1	Am	No	[68]
merobatzelladine A (**42**)	*Monanchora* sp.	Amami-Oshima Island, North Pacific Ocean	2009	1	Am	No	[50,69]
merobatzelladine B (**43**)	*Monanchora* sp.	Amami-Oshima Island, North Pacific Ocean	2009	1	Am	Yes	[69]
norbatzelladine A (**44**)	*Monanchora arbuscula*	Guadeloupe Island, North Atlantic Ocean	2009	3	Am, At	No	[68]
norbatzelladine L (**45**)	*Clathria calla*	Martinique, North Atlantic Ocean	2009	2	Am, At	No	[68]
dinorbatzelladine A (**46**)	*Monanchora arbuscula*	Guadeloupe island, North Atlantic Ocean	2009	3	Am, At	No	[68]
dinorbatzelladine B (**47**)	*Monanchora arbuscula*	Guadeloupe island, North Atlantic Ocean	2009	3	n.t.	No	[68]
dinordehydrobatzelladine B (**48**)	*Monanchora arbuscula*	Guadeloupe island, North Atlantic Ocean	2009	3	Am, At	No	[68]
dihomodehydrobatzelladine C (**49**)	*Monanchora arbuscula*	Guadeloupe island, North Atlantic Ocean	2009	2	Am, At	No	[68]
batzellamide A (**50**)	*Monanchora arbuscula*	Rio de Janeiro state, South Atlantic Ocean	2015	2	n.t.	No	[70]
hemibatzelladine J (**51**)	*Monanchora arbuscula*	Rio de Janeiro state, South Atlantic Ocean	2015	2	n.t.	No	[70]
Δ^19^-hemibatzelladine J (**52**)	*Monanchora arbuscula*	Rio de Janeiro state, South Atlantic Ocean	2015	2	n.t.	No	[70]
Δ^200^-hemibatzelladine J (**53**)	*Monanchora arbuscula*	Rio de Janeiro state, South Atlantic Ocean	2015	2	n.t.	No	[70]

Am, antimicrobial activity; Av, antiviral activity; At, antitumor activity; and n.t., not tested.

**Table 3:** GA classes.

No	Class 1	Class 2	Class 3	Class 4	Ref.
1	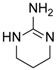	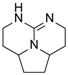	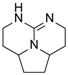 +1 or more guanidine moiety	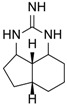	[70]
Sponges *	*Batzella* *Crambe* *Pseudaxinella* (*Ptilocaulis*)	*Clathria* *Crambe* *Hemimycale* *Monanchora* *Neofolitispa* *Ptilocaulis*	*Batzella* *Monanchora*	*Acanthella* *Arenochalina* *Batzella* *Biemna* *Clathria* *Monanchora* *Ptilocaulis*	-
2	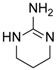	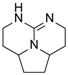	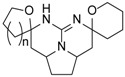 *n* = 1 or 3	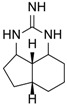	-
Sponges *	*Batzella* *Crambe* *Pseudaxinella* (*Ptilocaulis*)	*Batzella* *Clathria* *Monanchora*	*Crambe* *Hemimycale* *Monanchora* *Neofolitispa* *Ptilocaulis*	*Acanthella* *Arenochalina* *Batzella* *Biemna* *Clathria* *Monanchora* *Ptilocaulis*	-

* Sponges without any genus revision made.

**Table 4:** Characteristic TGA signals.

Atom Number	Crambescidin-Like GA Signals	Batzelladine-Like GA Signals
H_a_ *	dd from 2.8 to 2.5 ppm	m from 2.8 to 2.5 ppm
H_b_ *	m from 4.6 to 3.9 ppm	m from 4.6 to 3.9 ppm
H_c_ *	dt toward 4.3 ppm	m toward 4.3 ppm
H_d_ *	d from 3.5 to 2.9 ppm	dd from 3.5 to 2.9 ppm
H_4_ * et H_5_ * double bond	2 m toward 5.5 ppm	No signal

*: for a, b, c, d, 4 and 5 attributions, see above; d, doublet; dd, doublet of doublets; dt, doublet of triplets; m, multiplet.

**Table 5:** TGA Mass Spectrometry (MS) data.

Metabolites	*m/z* ([M + H]^+^ Unless Specified) and Δppm Found	Ref.
ptilomycalin A (**1**)	977.7915 (1.0 mmu) for the bis(trifluoroacetyl) derivative	[51]
crambescidin 800 (**2**)	801.6205 (1.3 mmu)	[55]
crambescidin 816 (**3**)	817.6151 (1.6 mmu)	[55]
crambescidin 830 (**4**)	831.6300 (2.3 mmu)	[55]
crambescidin 844 (**5**)	845.6471 (0.9 mmu)	[55]
13, 14, 15 -isocrambescidine 800 (**6**)	927.6521 (1.3 mmu) for the acetylated compound	[52]
crambidine (**7**)	967.6415 (6.8 mmu) for the acetylated compound	[52]
neofolitispate 1 (**8**)	686 (no HRMS data)	[57]
neofolitispate 2 (**9**)	672 (no HRMS data)	[57]
neofolitispate 3 (**10**)	658 (no HRMS data)	[57]
crambescidin 359 (**11**)	359.2567 (0.6 mmu)	[53]
crambescidin 431 (**12**)	431.2780 (0.4 mmu)	[53]
crambescidin 826 (**13**)	827.6389 (1.5 mmu)	[54]
crambescidin acid (**14**)	404.2541 (2.2 mmu)	[58]
crambescidic acid (**15**)	658.4781 (1.4 mmu)	[59]
16β-hydroxycrambescidin 359 (**16**)	376.2617 (1.7 mmu)	[73]
ptilomycalin D (**17**)	627.4994 *	[60]
monanchocidin (A) (**18**)	859.6267 (3.0 mmu)	[61]
monanchocidin B (**19**)	831.5978 (3.4 mmu)	[62]
monanchocidin C (**20**)	845.6150 (4.0 mmu)	[62]
monanchocidin D (**21**)	831.5920 (3.4 mmu)	[62]
monanchocidin E (**22**)	845.6120 (1.0 mmu)	[62]
monanchomycalin A (**23**)	813.6574 (0.2 mmu)	[63]
monanchomycalin B (**24**)	785.6259 (0.4 mmu)	[63]
monanchomycalin C (**25**)	813.6578 (0.3 mmu) and [M + 2H]^2+^ 407.3336 (0.7 mmu)	[64]
batzelladine A (**26**)	768.5839 (2.4 mmu)	[66]
batzelladine B (**27**)	738.5356 (3.8 mmu)	[66]
batzelladine C (**28**)	489.3903 (1.4 mmu)	[66]
batzelladine D (**29**)	463.3740 **	[66]
batzelladine E (**30**)	487.3728 (3.2 mmu)	[66]
batzelladine F (**31**)	624.5096 (0.6 mmu)	[67]
batzelladine G (**32**)	668,5353 (1.3 mmu)	[67]
batzelladine H (**33**)	609.4488 (0.4 mmu)	[67]
batzelladine I (**34**)		[67]
dehydrobatzelladine C (**35**)	487.3711 (4.9 mmu)	[53]
batzelladine J (**36**)	750.5361 (3.3 mmu)	[59]
batzelladine K (**37**)	250.2322 (3.9 mmu)	[73]
batzelladine L (**38**)	653.5458 (2.4 mmu) and [M + 2H]^2+^ 327.2798 (1.8 mmu)	[73]
batzelladine M (**39**)	[M + 2H]^2+^ 298.2399 (1.0 mmu)	[73]
batzelladine N (**40**)	[M + 2H]^2+^ 312.2546 (1.0 mmu)	[73]
clathriadic acid (**41**)	318.2173 (1.0 mmu)	[68]
merobatzelladine A (**42**)	360.3444 (6.6 mmu)	[69]
merobatzelladine B (**43**)	306.2909 (7.0 mmu)	[69]
norbatzelladine A (**44**)	754.5705 (0.7 mmu)	[68]
norbatzelladine L (**45**)	639.5327 (0.3 mmu)	[68]
dinorbatzelladine A (**46**)	740.5547 (0.9 mmu)	[68]
dinorbatzelladine B (**47**)	710.5074 (1.2 mmu)	[68]
dinordehydrobatzelladine B (**48**)	708.4919 (1.0 mmu)	[68]
dihomodehydrobatzelladine C (**49**)	515.4064 (1.3 mmu)	[68]
batzellamide A (**50**)	541.3878 (1.2 mmu)	[70]
hemibatzelladine J (**51**)	449.3238 (0.2 mmu)	[70]
Δ^19^-hemibatzelladine J (**52**)	447.3092 (0.8 mmu)	[70]
Δ^200^-hemibatzelladine J (**53**)		[70]

* Calculated *m/z* value: 626.4975; reported *m/z* value: 627.4994; ** not specified.

**Table 6:** Characteristic MS^2^ TGA signals.

*m/z* Fragment	*m/z* Fragment Loss	Fragment	Ref.
358 (or 359)		Crambescidin core	[53,54,55,59]
322 or 336 or 350		Batzelladine core + *n* = 6, 7 or 8 carbon side chain	[53,66,67,68,73]
114	113	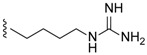	[53,59,66]
	101	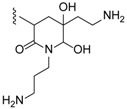	[61,62]
Intense 18	17	Carboxylic acid	-
Intense 48	47		

**Table 7:** TGA antiviral activities.

EC_50_ (µM Unless Specified)	HIV-1			HSV-1	HBV	Ref.
	Human PBMC	Envelope-Mediated Fusion	gp120 Binding to CD4			
ptilomycalin A (**1**)	0.011	n.t.	n.t.	0.25 *	n.t.	[51]
crambescidin 800 (**2**)	0.04	1–3	n.t.	1.25 µg/well ^a^	n.t.	[54,55,73]
crambescidin 816 (**3**)	n.t.	n.t.	n.t.	1.25 µg/well ^a^	n.t.	[55]
crambescidin 844 (**5**)	n.t.	n.t.	n.t.	1.25 µg/well ^a^	n.t.	[55]
13,14,15-isocrambescidin 800 (**6**)	n.t.	n.t.	n.t.	NA	n.t.	[56]
neofolitispate 1 (**8**)	n.t.	n.t.	n.t.	n.t.	7.4 **	[57]
neofolitispate 2 (**9**)	n.t.	n.t.	n.t.	n.t.		
neofolitispate 3 (**10**)	n.t.	n.t.	n.t.	n.t.		
crambescidin 826 (**13**)	n.t.	1–3	n.t.	n.t.	n.t.	[54]
batzelladine A (**26**)	n.t.	n.t.	29	n.t.	n.t.	[66]
batzelladine B (**27**)	n.t.	n.t.	31	n.t.	n.t.	[66]
batzelladine C (**28**)	7.7	n.t.	n.t.	n.t.	n.t.	[73]
batzelladine D (**29**)	n.t.	n.t.	72	n.t.	n.t.	[66]
dehydrobatzelladine C (**35**)	5.5	n.t.	n.t.	n.t.	n.t.	[73]
batzelladine L (**38**)	1.6	n.t.	n.t.	n.t.	n.t.	[73]
batzelladine M (**39**)	7.7	n.t.	n.t.	n.t.	n.t.	[73]
batzelladine N (**40**)	2.4	n.t.	n.t.	n.t.	n.t.	[73]

HIV, Human immunodeficiency virus; PBMC, Peripheral Blood Mononuclear cells; gp, glycoprotein; HSV, *Herpes simplex* virus; HBV, Human hepatitis B virus; n.t., not tested, NA, not active; * converted from µM to µg/mL; ** calculated from compound (**9**) molecular weight; ^a^ Diffuse cytotoxicity at 1.25 µg/well.

**Table 8:** TGA antimicrobial activities.

IC_50_ (Values are Expressed in µg/mL Unless Specified)	Bacteria						Yeast	Fungi		Parasites							Ref.
	*S. aureus*	*MRSA*	*P. aeruginosa*	*M. tuberculosis*	*M. intracellulare*	*V. anguillarum*	*C. albicans*	*C. neoformans*	*A. fumigatus (AC)*	*P. falciparum*			*L. infatum*	*L. donovani*	*T. cruzi*	*T. brucei brucei*	
										D6 Clone	W2 Clone	FcB1					
ptilomycalin A (**1**)	0.25	0.30	1.0	>128	10	n.t.	0.15	0.10	1.25	0.12	0.11	0.08 *	n.t.	5.9	n.t.	n.t.	[68,73]
crambescidin 800 (**2**)	0.20	0.35	0.95	46.5	15	n.t.	0.15	0.10	1.25	0.11	0.13	n.t.	n.t.	6.80	n.t.	n.t.	[73]
16β-hydroxycrambescidin 800 (**16**)	NA	NA	NA	>128	NA	n.t.	NA	NA	NA	3.8	NA	NA	n.t.	n.t.	n.t.	n.t.	[73]
batzelladine A (**26**)	n.t.	n.t.	n.t.	n.t.	n.t.	n.t.	n.t.	n.t.	n.t.	n.t.	n.t.	0.2*	n.t.	n.t.	n.t.	n.t.	[68]
batzelladine C (**28)**	0.20	0.30	10	34.7	0.9	n.t.	0.90	0.40	5.0	0.09	0.11	n.t.	n.t.	5.5	n.t.	n.t.	[73]
batzelladine D (**29**)	n.t.	n.t.	n.t.	n.t.	n.t.	n.t.	n.t.	n.t.	n.t.	n.t.	n.t.	n.t.	0.9 *	n.t.	29 *	n.t.	[70]
batzelladine F (**31**)	n.t.	n.t.	n.t.	n.t.	n.t.	n.t.	n.t.	n.t.	n.t.	n.t.	n.t.	n.t.	2.5 *	n.t.	3.1 *	n.t.	[70]
dehydrobatzelladine C (**35**)	0.40	0.70	NA.	37.7	1.0	n.t.	1.0	0.6	20	0.073	0.13	n.t.	n.t.	5.70	n.t.	n.t.	[73]
batzelladine L (**38**)	0.35	0.40	3.50	1.68	0.25	n.t.	0.40	0.55	2.5	0.073	0.10	0.2 *	1.3 *	1.90	1.3 *	n.t.	[68,73]
batzelladine M (**39**)	3.0	5.0	NA	28.5	3.50	n.t.	6.0	8.0	NA	0.21	0.27	n.t.	n.t.	8.50	n.t.	n.t.	[73]
batzelladine N (**40**)	n.t.	n.t.	n.t.	3.18	n.t.	n.t.	n.t.	n.t.	n.t.	n.t.	n.t.	n.t.	n.t.	n.t.	n.t.	n.t.	[73]
clathriadic acid (**41**)	n.t.	n.t.	n.t.	n.t.	n.t.	n.t.	n.t.	n.t.	n.t.	n.t.	n.t.	1.4 *	n.t.	n.t.	n.t.	n.t.	[68]
merobatzelladine A (**42**)	n.t.	n.t.	n.t.	n.t.	n.t.	a	n.t.	n.t.	n.t.	0.48			n.t.	n.t.	n.t.	0.24	[69]
merobatzelladine B (**43**)	n.t.	n.t.	n.t.	n.t.	n.t.		n.t.	n.t.	n.t.	0.97			n.t.	n.t.	n.t.	0.24	[69]
norbatzelladine A (**44**)	n.t.	n.t.	n.t.	n.t.	n.t.	n.t.	n.t.	n.t.	n.t.	n.t.	n.t.	0.2 *	n.t.	n.t.	n.t.	n.t.	[68]
norbatzelladine L (**45**)	n.t.	n.t.	n.t.	n.t.	n.t.	n.t.	n.t.	n.t.	n.t.	n.t.	n.t.	0.3 *	1.3 *	n.t.	4.4 *	n.t.	[68,70]
dinorbatzelladine A (**46**)	n.t.	n.t.	n.t.	n.t.	n.t.	n.t.	n.t.	n.t.	n.t.	n.t.	n.t.	1.7 *	n.t.	n.t.	n.t.	n.t.	[68]
dinordehydrobatzelladine B (**48**)	n.t.	n.t.	n.t.	n.t.	n.t.	n.t.	n.t.	n.t.	n.t.	n.t.	n.t.	0.6 *	n.t.	n.t.	n.t.	n.t.	[68]
dihomodehydrobatzelladine C (**49**)	n.t.	n.t.	n.t.	n.t.	n.t.	n.t.	n.t.	n.t.	n.t.	n.t.	n.t.	2.3 *	n.t.	n.t.	n.t.	n.t.	[68]

*S. aureus*, *Staphyloccocus aureus*; MRSA, Methicilline resistant *Staphyloccocus aureus*; *P. aeruginosa*, *Pseudomonas aeruginosa*; *M. tuberculosis*, *Mycobacterium tuberculosis*; *M. intracellulare*, *Mycobacterium intracellulare*; *V. angillarum*, *Vibrio angillarum*; *C. albicans*, *Candida albicans*; *A. fumigatus*, *Aspergillus fumigatus*; *P. falciparum*, *Plasmodium falciparum*; *L. infatum*, *Leishmania infatum*; *L. donovani*, *Leishmania donovani*; *T. cruzi*, *Trypanosoma cruzi*; *T. brucei brucei*, *Trypanosoma brucei brucei*; AC, active concentration; NA, not active according to the authors; n.t., not tested; * converted from µM to µg/mL; a, 9–10 mm/50 µg.

**Table 9:** TGA antitumor activities (values are expressed in µg/mL unless specified).

		Prostate	Ovary	Breast		Melanoma	Lung	Leukemia			Pancreas	Colon				Cervix	Ref.
		DU-145	IGROV	SK-BR3	MDA-MB-231	SK-MEL-28	NSCL A549	L-562	HL-60	THP-1	PANCL	HT29	HCT-16	LOVO	LOVO-DOX	HeLa	
ptilomycalin A (**1**)	GI_50_	0.05	0.04	0.07	n.t.	0.03	0.08	0.04	n.t.	n.t.	0.04	0.03	n.t.	0.05	0.05	0.04	[73]
	TGI	1.22	1.74	0.54	n.t.	0.11	1.23	1.33	n.t.	n.t.	0.99	0.19	n.t.	2.14	2.04	0.22	
	LC_50_	5.21	n.t.	n.t.	n.t.	0.98	9.79	9.72	n.t.	n.t.	0.98	5.37	n.t.	9.79	8.48	3.21	
crambescidin 800 (**2**)	GI_50_	0.19	0.05	0.16	n.t.	0.04	0.11	0.02	n.t.	n.t.	0.04	0.04	n.t.	0.08	0.08	0.05	[73]
	TGI	1.38	2.50	0.56	n.t.	0.11	1.36	0.06	n.t.	n.t.	1.53	0.23	n.t.	2.29	2.02	0.21	
	LC_50_	7.01	n.t.	n.t.	n.t.	1.70	9.68	6.73	n.t.	n.t.	8.66	5.75	n.t.	8.97	8.50	1.58	
crambescidin 816 (**3**)	GI_50_	n.t.	n.t.	n.t.	n.t.	n.t.	n.t.	n.t.	n.t.	n.t.	n.t.	n.t.	IC_50_ 0.24	n.t.	n.t.	n.t.	[52]
	TGI	n.t.	n.t.	n.t.	n.t.	n.t.	n.t.	n.t.	n.t.	n.t.	n.t.	n.t.		n.t.	n.t.	n.t.	
	LC_50_	n.t.	n.t.	n.t.	n.t.	n.t.	n.t.	n.t.	n.t.	n.t.	n.t.	n.t.		n.t.	n.t.	n.t.	
monanchocidin A (**18**)	GI_50_	n.t.	n.t.	n.t.	n.t.	n.t.	n.t.	n.t.	n.t.	IC_50_ 4.4 *	n.t.	n.t.	n.t.	n.t.	n.t.	IC_50_ 10.1 *	[61]
	TGI	n.t.	n.t.	n.t.	n.t.	n.t.	n.t.	n.t.	n.t.		n.t.	n.t.	n.t.	n.t.	n.t.		
	LC_50_	n.t.	n.t.	n.t.	n.t.	n.t.	n.t.	n.t.	n.t.		n.t.	n.t.	n.t.	n.t.	n.t.		
monanchocidin B (**19**)	GI_50_	n.t.	n.t.	n.t.	n.t.	n.t.	n.t.	n.t.	IC_50_ 0.17 *	n.t.	n.t.	n.t.	n.t.	n.t.	n.t.	n.t.	[62]
	TGI	n.t.	n.t.	n.t.	n.t.	n.t.	n.t.	n.t.		n.t.	n.t.	n.t.	n.t.	n.t.	n.t.	n.t.	
	LC_50_	n.t.	n.t.	n.t.	n.t.	n.t.	n.t.	n.t.		n.t.	n.t.	n.t.	n.t.	n.t.	n.t.	n.t.	
monanchocidin C (**20**)	GI_50_	n.t.	n.t.	n.t.	n.t.	n.t.	n.t.	n.t.	IC_50_ 0.09 *	n.t.	n.t.	n.t.	n.t.	n.t.	n.t.	n.t.	[62]
	TGI	n.t.	n.t.	n.t.	n.t.	n.t.	n.t.	n.t.		n.t.	n.t.	n.t.	n.t.	n.t.	n.t.	n.t.	
	LC_50_	n.t.	n.t.	n.t.	n.t.	n.t.	n.t.	n.t.		n.t.	n.t.	n.t.	n.t.	n.t.	n.t.	n.t.	
monanchocidin D (**21**)	GI_50_	n.t.	n.t.	n.t.	n.t.	n.t.	n.t.	n.t.	IC_50_ 0.69 *	n.t.	n.t.	n.t.	n.t.	n.t.	n.t.	n.t.	[62]
	TGI	n.t.	n.t.	n.t.	n.t.	n.t.	n.t.	n.t.		n.t.	n.t.	n.t.	n.t.	n.t.	n.t.	n.t.	
	LC_50_	n.t.	n.t.	n.t.	n.t.	n.t.	n.t.	n.t.		n.t.	n.t.	n.t.	n.t.	n.t.	n.t.	n.t.	
monanchocidin E (**22**)	GI_50_	n.t.	n.t.	n.t.	n.t.	n.t.	n.t.	n.t.	IC_50_ 0.55 *	n.t.	n.t.	n.t.	n.t.	n.t.	n.t.	n.t.	[62]
	TGI	n.t.	n.t.	n.t.	n.t.	n.t.	n.t.	n.t.		n.t.	n.t.	n.t.	n.t.	n.t.	n.t.	n.t.	
	LC_50_	n.t.	n.t.	n.t.	n.t.	n.t.	n.t.	n.t.		n.t.	n.t.	n.t.	n.t.	n.t.	n.t.	n.t.	
monanchomycalin A (**23**)	GI_50_	n.t.	n.t.	n.t.	n.t.	n.t.	n.t.	n.t.	IC_50_ 0.10 *	n.t.	n.t.	n.t.	n.t.	n.t.	n.t.	n.t.	[63]
	TGI	n.t.	n.t.	n.t.	n.t.	n.t.	n.t.	n.t.		n.t.	n.t.	n.t.	n.t.	n.t.	n.t.	n.t.	
	LC_50_	n.t.	n.t.	n.t.	n.t.	n.t.	n.t.	n.t.		n.t.	n.t.	n.t.	n.t.	n.t.	n.t.	n.t.	
monanchomycalin B (**24**)	GI_50_	n.t.	n.t.	n.t.	n.t.	n.t.	n.t.	n.t.	IC_50_ 0.11 *	n.t.	n.t.	n.t.	n.t.	n.t.	n.t.	n.t.	[63]
	TGI	n.t.	n.t.	n.t.	n.t.	n.t.	n.t.	n.t.		n.t.	n.t.	n.t.	n.t.	n.t.	n.t.	n.t.	
	LC_50_	n.t.	n.t.	n.t.	n.t.	n.t.	n.t.	n.t.		n.t.	n.t.	n.t.	n.t.	n.t.	n.t.	n.t.	
batzelladine C (**28**)	GI_50_	0.68	0.81	0.66	n.t.	1.45	1.40	0.62	n.t.	n.t.	0.55	0.65	n.t.	2.06	2.25	0.70	[73]
	TGI	2.27	3.43	2.89	n.t.	3.67	3.42	3.01	n.t.	n.t.	2.03	2.16	n.t.	4.37	4.72	2.22	
	LC_50_	0.69	n.t.	n.t.	n.t.	9.24	8.31	n.t.	n.t.	n.t.	7.14	6.70	n.t.	9.29	0.99	6.50	
dehydrobatzelladine C (**35**)	GI_50_	0.46	0.73	0.23	n.t.	0.89	1.19	0.48	n.t.	n.t.	0.43	0.48	n.t.	1.60	2.07	0.48	[73]
	TGI	1.91	4.17	1.14	n.t.	3.48	3.24	2.42	n.t.	n.t.	1.83	1.77	n.t.	3.68	4.48	1.52	
	LC_50_	7.15	n.t.	n.t.	n.t.	n.t.	8.81	n.t.	n.t.	n.t.	8.66	7.50	n.t.	8.47	9.69	5.45	
batzelladine L (**38**)	GI_50_	0.44	0.52	0.23	n.t.	0.88	1.30	n.t.	n.t.	n.t.	0.34	4.96	n.t.	1.09	n.t.	0.38	[73]
	TGI	1.39	1.74	0.56	n.t.	2.18	9.99	n.t.	n.t.	n.t.	1.33	n.t.	n.t.	2.41	n.t.	1.16	
	LC_50_	3.78	5.01	2.10	n.t.	4.95	n.t.	n.t.	n.t.	n.t.	4.38	n.t.	n.t.	5.36	n.t.	3.58	
batzelladine M (**39**)	GI_50_	1.77	2.28	1.12	n.t.	1.18	3.80	n.t.	n.t.	n.t.	1.22	3.56	n.t.	1.99	n.t.	1.64	[73]
	TGI	3.44	5.08	2.51	n.t.	4.66	n.t.	0.00	n.t.	n.t.	3.58	n.t.	n.t.	3.56	n.t.	3.05	
	LC_50_	6.66	n.t.	5.59	n.t.	n.t.	n.t.	0.00	n.t.	n.t.	n.t.	n.t.	n.t.	6.37	n.t.	5.68	
batzelladine N (**40**)	GI_50_	1.39	1.78	1.12	n.t.	1.47	1.94	0.66	n.t.	n.t.	1.37	1.31	n.t.	1.96	4.42	0.59	[73]
	TGI	3.12	4.97	3.84	n.t.	3.41	4.29	3.27	n.t.	n.t.	3.50	3.11	n.t.	4.16	n.t.	1.80	
	LC_50_	7.04	n.t.	n.t.	n.t.	7.97	9.47	n.t.	n.t.	n.t.	8.97	7.35	n.t.	8.85	n.t.	5.13	
clathriadic acid (**41**)	GI_50_	n.t.	n.t.	n.t.	13.5	n.t.	>30	n.t.	n.t.	n.t.	n.t.	>30	n.t.	n.t.	n.t.	n.t.	[68]
	TGI	n.t.	n.t.	n.t.	>30	n.t.	>30	n.t.	n.t.	n.t.	n.t.	>30	n.t.	n.t.	n.t.	n.t.	
	LC_50_	n.t.	n.t.	n.t.	>30	n.t.	>30	n.t.	n.t.	n.t.	n.t.	>30	n.t.	n.t.	n.t.	n.t.	
norbatzelladine A (**44**)	GI_50_	n.t.	n.t.	n.t.	3.8	n.t.	2.1	n.t.	n.t.	n.t.	n.t.	1.6	n.t.	TC_50_ 4.7 µM	n.t.	n.t.	[68]
	TGI	n.t.	n.t.	n.t.	6.4	n.t.	4.6	n.t.	n.t.	n.t.	n.t.	3.2	n.t.		n.t.	n.t.	
	LC_50_	n.t.	n.t.	n.t.	11.4	n.t.	8.6	n.t.	n.t.	n.t.	n.t.	5.7	n.t.		n.t.	n.t.	
norbatzelladine L (**45**)	GI_50_	n.t.	n.t.	n.t.	0.7	n.t.	1.1	n.t.	n.t.	n.t.	n.t.	1.9	n.t.	n.t.	n.t.	n.t.	[68]
	TGI	n.t.	n.t.	n.t.	1.9	n.t.	2.1	n.t.	n.t.	n.t.	n.t.	4.2	n.t.	n.t.	n.t.	n.t.	
	LC_50_	n.t.	n.t.	n.t.	4.8	n.t.	4.2	n.t.	n.t.	n.t.	n.t.	7.6	n.t.	n.t.	n.t.	n.t.	
dinorbatzelladine A (**46**)	GI_50_	n.t.	n.t.	n.t.	3.0	n.t.	1.9	n.t.	n.t.	n.t.	n.t.	1.9	n.t.	n.t.	n.t.	n.t.	[68]
	TGI	n.t.	n.t.	n.t.	3.8	n.t.	4.2	n.t.	n.t.	n.t.	n.t.	4.2	n.t.	n.t.	n.t.	n.t.	
	LC_50_	n.t.	n.t.	n.t.	5.4	n.t.	7.6	n.t.	n.t.	n.t.	n.t.	7.6	n.t.	n.t.	n.t.	n.t.	
dinordehydro-batzelladine B (**48**)	GI_50_	n.t.	n.t.	n.t.	n.t.	n.t.	7.9	n.t.	n.t.	n.t.	n.t.	6.2	n.t.	n.t.	n.t.	n.t.	[68]
	TGI	n.t.	n.t.	n.t.	n.t.	n.t.	>14	n.t.	n.t.	n.t.	n.t.	> 14	n.t.	n.t.	n.t.	n.t.	
	LC_50_	n.t.	n.t.	n.t.	n.t.	n.t.	>14	n.t.	n.t.	n.t.	n.t.	> 14	n.t.	n.t.	n.t.	n.t.	
dihomodehydro-batzelladine C (**49**)	GI_50_	n.t.	n.t.	n.t.	6.1	n.t.	>30	n.t.	n.t.	n.t.	n.t.	>30	n.t.	n.t.	n.t.	n.t.	[68]
	TGI	n.t.	n.t.	n.t.	9.8	n.t.	>30	n.t.	n.t.	n.t.	n.t.	>30	n.t.	n.t.	n.t.	n.t.	
	LC_50_	n.t.	n.t.	n.t.	15.6	n.t.	>30	n.t.	n.t.	n.t.	n.t.	>30	n.t.	n.t.	n.t.	n.t.	

n.t., not tested; * calculated value from µM to µg/mL.

## Supplementary Files

Supplementary File 1

## Abbreviations

The following abbreviations are used in this manuscript:

A.m.	Antimicrobial activity
A.v.	Antiviral activity
A.t.	Antitumor activity
COSY	Correlation Spectroscopy
EC_50_	half maximal Effective Concentration
GA	Guanidine Alkaloids
GI_50_	half maximal Growth Inhibition
gp120	glycoprotein 120
HBV	Human hepatitis B virus
HIV-1	Human Immunodeficiency Virus 1
HSV-1	*Herpes simplex* Virus 1
IC_50_	half maximal Inhibitory Concentration
LC_50_	half maximal Lethal Concentration
MIC	Minimum Inhibitory Concentration
MS	Mass Spectrometry
NMR	Nuclear Magnetic Resonance
n.t.	Not tested
PBMC	Peripheral Blood Mononuclear Cells
SM	Secondary Metabolites
TC_50_	half maximal Toxic Concentration
TGA	Triazaacenaphthylene Guanidine Alkaloids
TGI	Total Growth Inhibition concentration
