# Supplementary material for: Polycyclic Guanidine Alkaloids from Poecilosclerida Marine Sponges
Source: Mar Drugs. 2016 Apr 9;14(4):77. doi: 10.3390/md14040077 (PMC4849081; doi:10.3390/md14040077)
Supplement: Supplementary File 1 [file marinedrugs-14-00077-s001.zip › Daniel Coaten - Letter of confirmation.pdf]

Keilir Institute of Technology

Daniel Coaten
Keilir Institute of Technology
Tel: +354 578 4082
Email: daniel@keilir.net

MDPI AG
Marine Drugs Editorial Office
Klybeckstrasse 64,
4057 Basel,
Switzerland

25/03/16

Re: Letter of confirmation.

To whom it may concern.

Dear Sir/Madam,

Through my capacity as native English speaker and senior lecturer of English composition and technical writing courses based at Keilir Institute of Technology (validated by the University of Iceland), I hereby confirm that I have read through the latest version of the manuscript entitled “Polycyclic Guanidine Alkaloids from Poecilosclerida Marine Sponges” (updated on the 25/03/2016).

To the best of my knowledge, I found that the written English, grammar and style contained within this document to be of a satisfactory level for which it is intended.

Yours faithfully,

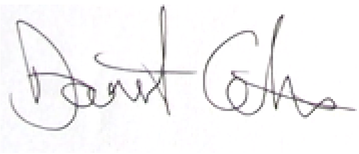

Daniel Coaten
Assistant Professor

Grænásbraut 910, 235 Reykjanesbær • Tel: 578 4000 • Fax: 578 4001 • keilir@keilir.net • www.keilir.net
