# Supplementary figures and images for: Polycyclic Guanidine Alkaloids from Poecilosclerida Marine Sponges
Source: Mar Drugs. 2016 Apr 9;14(4):77. doi: 10.3390/md14040077 (PMC4849081; doi:10.3390/md14040077)

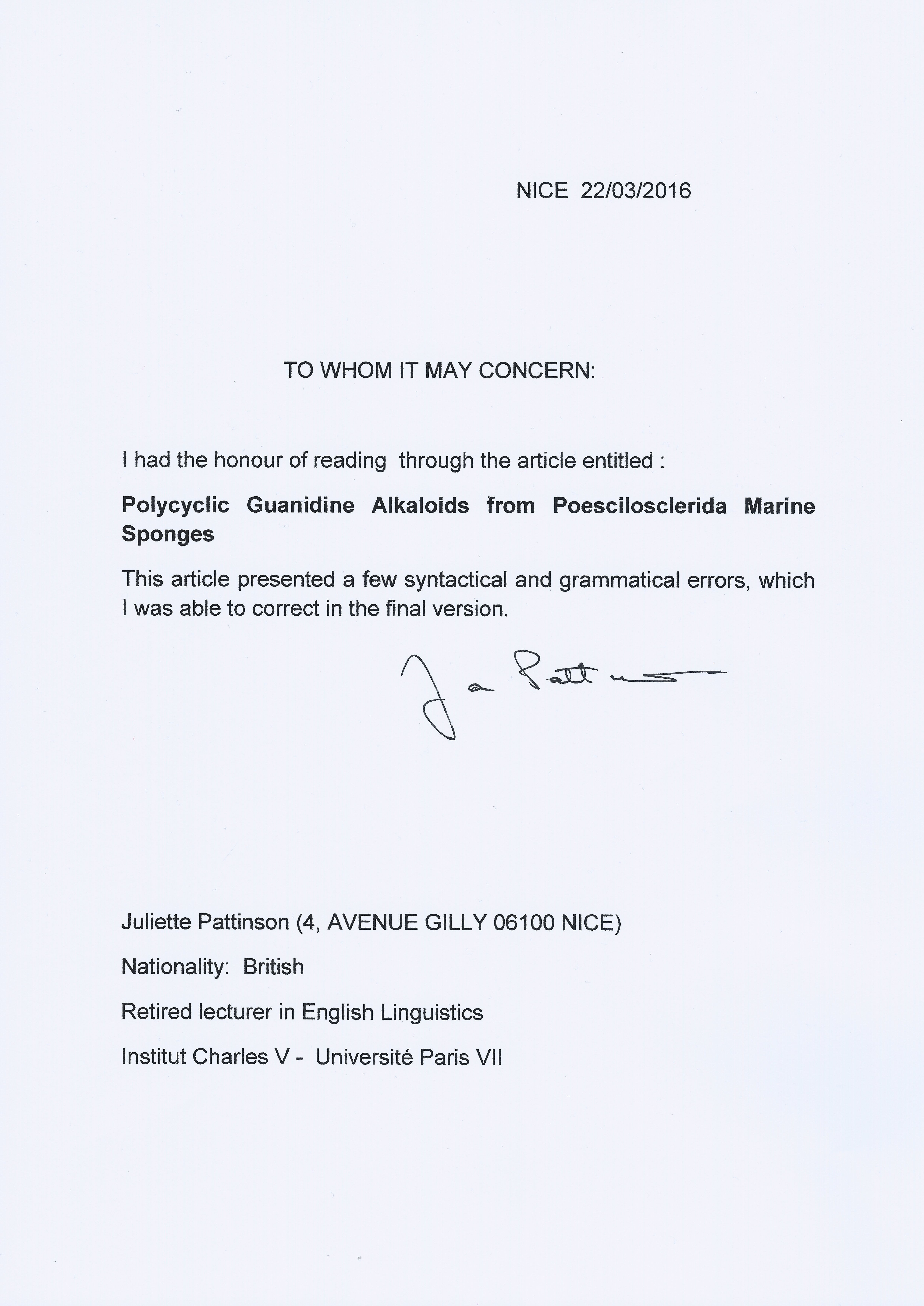

Supplement: Supplementary File 1 [file marinedrugs-14-00077-s001.zip › SCAN1130.JPG]

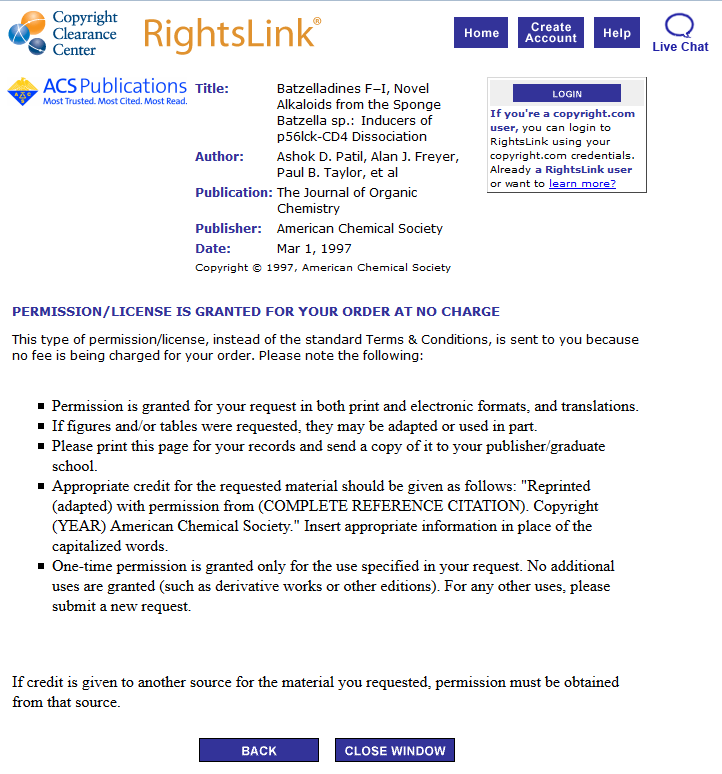

Supplement: Supplementary File 1 [file marinedrugs-14-00077-s001.zip › Permission to use figure 11.png]
